# Annotations

**Published:** 1911-04-08

**Authors:** 


### Annotations.

### The Florence Nightingale Memorial.

We should like to see the medical profession and all hospital workers unite in giving a contribution to this Memorial Fund. This is one of those memorials the cost of which should be met by the spontaneous gifts of hospital workers arranged in brigades, the unit being the hospital or institution to which the workers are attached. Florence Nightingale revolutionised the provision for, and the nursing of, the sick. She contributed a great deal to the development of the modern hospital as it exists to-day. Every real hospital worker must recognise the momentous importance of her work, example, and spirit in the evolution of the modern hospital, and regard it as a privilege to add his mite to the fund which will give the nation a permanent memorial. Why should not every hospital, large and small, *i.e.* every agency for the relief of the sick, throughout the United Kingdom, start a Shilling Fund and send the proceeds to the Treasurer, Mr. J. G. Wainwright, at St. Thomas's Hospital? The individual subscription should be small and uniform, so that all workers may join on the same basis, and no hospital can be properly represented except by the unity of all its workers. If this suggestion is taken up it will constitute the most magnificent form of memorial which it is possible to conceive. Every institution for the relief of mental or bodily illness ought to be included. Such a movement would worthily perpetuate the influence for good which Florence Nightingale exercised all over the world, especially in this kingdom and empire. The organisation is simple. Let the chairman receive the shillings of the Committee; the secretary to the medical staff their contributions; and the superintendent and matron those of all other workers attached to each institution—and the thing is done.

### London's Necessity: One Great Medical School.

We have always insisted that London is sadly behind other great centres in regard to medical education, owing to the existence of a number of competing medical schools which have handicapped the development of this branch of instruction and kept the Metropolis from being, as it should be, the centre and heart of medical study and instruction for students throughout the Empire. A perusal of the report of the Distribution Committee of the King's Fund on St. George's Hospital proves that the present plan of maintaining a number of small competing medical schools in London is unnecessarily expensive and surrounded by difficulties and disadvantages which call for a reconsideration of the whole position of medical education in London. Those who have the interests of the medical profession in this country at heart, and who desire to see that the medical education shall be placed upon a sufficiently strong and enduring basis in London to make it adequate to fulfil worthily the responsibilities of those entrusted with the education and training of medical students, will agree that the present evils can only be removed and the future of medical education properly conserved by securing one great medical school in London with ample funds to provide that, as a teaching centre and a centre of scientific medicine and surgery at its best, it shall possess no rival in the civilised world. We hope, therefore, that one effect of the present controversy and a close study of the very able report of the Distribution Committee on the relations of St. George's Hospital to its medical school will be to stir up medical and public opinion sufficiently to ensure the appointment of a Commission to inquire into the whole question of medical education in London with a view to the establishment of one great medical school.

### A Note on Poor Law Reform.

The publication of a new edition of the Right Hon. Charles Booth's work on "Poor Law Reform" comes opportunely at this moment. But additional importance and interest are lent to this publication by the inclusion of a comment by Sir Arthur Downes, who sounds a note of warning against the danger of assuming that the existing machinery of the Poor Law must of necessity be "broken up and replaced by a revolutionary embarkation on untried experiment." He is of opinion that advance would be safer and more sure by reform as opposed to revolution, and advises strongly against the hasty acceptance of the new scheme of the County Councils' Association, a scheme which has yet to be endorsed by the county councils themselves. To quote the postscript, this scheme "does not solve the problem of the great urban populations where need for reform is greatest, while it threatens a maximum of disturbance to the rural districts where need is least pressing." Stress is laid on the experience that classification can only be effectually carried out in the hands of a single authority, and it is pointed out that the alternatives of the Minority Report and of the County Councils' Association "are in reality a classification of authorities which would tend only to complicate the sorting out of the persons dealt with by them." The proposals of Mr. Booth were put forward only as tentative suggestions, though some have considered them as representing a hard-and-fast scheme of settled detail. The essence of his proposals was the grouping together of units of administrative areas for such general or special purposes as local needs might require. Sir Arthur Downes refers to the example of the Metropolitan Asylums Board as an excellent one, quoting its admirable care and classification of four thousand Poor Law children and its satisfactory financial position. He attributes the good results to the "advantage which a single authority for various classes of public assistance has in adapting its accommodation to the special needs the occasion may require, thereby avoiding waste." The conclusion arrived at is that a scheme such as contemplated by the County Councils' Association would be found in practice both ineffective and productive of friction, while the framework which Mr. Booth has designed is one which might be adjusted and filled in to any desired degree.

